# The Balance between Plasmacytoid DC versus Conventional DC Determines Pulmonary Immunity to Virus Infections

**DOI:** 10.1371/journal.pone.0001720

**Published:** 2008-03-05

**Authors:** Joost J. Smit, Dennis M. Lindell, Louis Boon, Mirjam Kool, Bart N. Lambrecht, Nicholas W. Lukacs

**Affiliations:** 1 Department of Pathology, University of Michigan Medical School, Ann Arbor, Michigan, United States of America; 2 Bioceros BV, Utrecht, The Netherlands; 3 Department of Pulmonary Medicine, Erasmus University Medical Center, Rotterdam, The Netherlands; Instituto Oswaldo Cruz and FIOCRUZ, Brazil

## Abstract

**Background:**

Respiratory syncytial virus (RSV) infects nearly all infants by age 2 and is a leading cause of bronchiolitis. RSV may employ several mechanisms to induce immune dysregulation, including dendritic cell (DC) modulation during the immune response to RSV.

**Methods and Findings:**

Expansion of cDC and pDC by Flt3L treatment promoted an anti-viral response with reduced pathophysiology characterized by decreased airway hyperreactivity, reduced Th2 cytokines, increased Th1 cytokines, and a reduction in airway inflammation and mucus overexpression. These protective aspects of DC expansion could be completely reversed by depleting pDCs during the RSV infection. Expansion of DCs by Flt3L treatment enhanced in CD8+ T cell responses, which was reversed by depletion of pDC.

**Conclusions:**

These results indicate that a balance between cDC and pDC in the lung and its lymph nodes is crucial for the outcome of a pulmonary infection. Increased pDC numbers induced by Flt3L treatment have a protective impact on the nature of the overall immune environment.

## Introduction

Respiratory syncytial virus (RSV) infects nearly all infants by age 2 and is the leading cause of bronchiolitis in children worldwide. It is estimated by the CDC that up to 125,000 pediatric hospitalizations in the United States each year are due to RSV, at an annual cost of over $300,000,000 [1]. While RSV is especially detrimental in very young infants whose airways are small and easily occluded, RSV is also widely becoming recognized as an important pathogen in the elderly, transplant recipients, patients with chronic obstructive pulmonary disease (COPD), as well as chronic asthmatics [2]. Despite the generation of RSV-specific adaptive immune responses, RSV does not confer protective immunity and recurrent infections throughout life are common [3]. This suggests that RSV may employ several mechanisms to induce immune dysregulation, illustrated by the failure of a vaccine to RSV in the 60′s [4]. It has been suggested that the nature of the immune response is dependent upon the characteristics of the innate immune system and APC function. Our present study was aimed at determining whether the balance of DC subsets or the merely increased numbers of DCs were the key to effective anti-viral immune responses.

Two major DCs subsets in the mouse are CD11b+, CD11c+ myeloid/conventional DC (mDC/cDC) and CD11b-, B220+ plasmacytoid DC (pDC) [5]. A series of studies have outlined distinct differences in the ability of DC subsets to induce immune responses. In particular, cDC have been implicated in driving a pro-allergic response, while pDC have been identified to block or “tolerize” the pulmonary immune environment against Th2 responses [6]. Other data have identified that pDC can participate or functionally mediate anti-viral responses in the lung, either directly by secretion of IFN-α or indirectly through activation of other cell populations [7], [8]. Previously, it was demonstrated that treating animals with recombinant Flt3 ligand (FLt3L) increased numbers of cDC and pDC in circulation as well as in peripheral organs, which can protect animals from metastatic tumor development and prolong survival, and reverse late stage allergic responses in the lung [9]–[13]. Previous results from our laboratory have indicated that depletion of pDC from the lungs of RSV-infected mice results in a more pathologic response with increased AHR, mucus, and Th2 cytokine profile [14]. In the present study we used Flt3L treatment *in vivo* before RSV infection to increase the numbers of both cDC and pDC to facilitate enhanced innate immune responses. Coupled with pDC depletion, we have been able to establish a differential role of cDC and pDC for maintenance of the appropriate anti-viral pulmonary immune environment. Although we were able to maintain cDC numbers upon pDC depletion, this increase was not able to maintain the enhanced anti-viral pulmonary environment, rather it increased the severity of the pathophysiologic responses. Thus, these studies further establish the differential roles of pDC and cDC subsets within the lung during viral infections.

## Materials and Methods

### Animals

Female BALB/cByJ mice, 6–8 weeks of age, were obtained from the Jackson laboratory. All mice were housed under specific pathogen-free conditions within the animal care facility at the University of Michigan. The University of Michigan Committee on the Use and Care of Animals approved all experiments.

### Culture and stimulation of mouse and human cDC and pDC

For culture of pDC and cDC, mouse bone marrow was isolated and cultured with Flt3L (200 ng/ml, Amgen) or with GM-CSF (10 ng/ml, R&D) respectively, as previously described [15] [16]. Plasmacytoid DC were isolated from the Flt3L culture after 9 days using a MACS bead pDC isolation kit (Miltenyi) or by cell sorting for CD11c^+^/B220^+^ cells. After isolation, cells were stimulated with RSV (MOI of 2) for 24 h. Cells were stained with the following antibodies: anti-mouse I-Ab, CD86, CD80, CD40, (BD Pharmingen). Supernatants were frozen until further analysis.

### Expansion of DC, RSV infection model and RSV plaque assay

Mice were treated with human Flt3L (kindly provided by Amgen, Seattle, USA) for 10 consecutive days to expand DC *in vivo*, as described before [9]. RSV subtype A, Umich/line 19 strain was derived from a clinical isolate at the University of Michigan and was propagated in Hep2 cells. Infection was allowed to proceed until syncytia were observed. Then, cells were frozen at −80°C and the supernatants were harvested, clarified, and aliquoted. This RSV preparation was tested negative for both endotoxin and mycoplasma. To determine viral titers in culture supernatants and lung homogenates, an immunoplaque assay was performed as previously described [14]. For RSV infection, mice were anesthetized with ketamine/xylazine and intratracheally infected with ∼1×10^5^ PFU of RSV. Administration of UV irradiated RSV does not elicit any responses in the lungs in mice, demonstrating no confounding effects of the medium used to culture RSV *in vivo*. Plasmacytoid DC were depleted *in vivo* by i.p. injecting mice 1 day before and 1 and 3 days after RSV infection with 150 µg 120G8 antibody as described before [14]. All control and RSV-infected mice received an isotype control antibody (rat IgG, Sigma).

### Assessment of lung pathology

Eight days after RSV infection, airway reactivity in anesthetized mice was measured as previously described [14], [17]–[20]. For this purpose, mice were anesthetized with sodium pentobarbital, the trachea was cannulated and ventilated using a pump ventilator. After baseline measurements, mice were injected i.v. with 2.5 µg of methacholine (125 mg/kg; Sigma-Aldrich) and the peak airway resistance was recorded. The optimal dose was determined in uninfected mice using single increasing doses of methacholine in different mice to choose the highest dose that gave little responsiveness. This dose was then used in RSV infected mice in the remainder of the mice, as previously described.

Lungs were inflated and maintained in formalin for 24 h before being processed into paraffin using standard histological techniques. Lung tissue sections were stained with hematoxylin and eosin (H&E) for analysis of inflammatory cell accumulation and alcian blue/periodic acid-Schiff for assessment of mucus production. To quantify the mucus production in the lung, PAS sections were randomized and examined and scored on a scale from 1 to 4, with 1 representing no mucus cell content and 4 representing airways filled with mucus.

Single cells suspensions of lung draining lymph nodes were prepared by isolation of mediastinal lymph nodes, pushing cells trough a nylon mesh using a syringe and lysis of red blood cells. Cells were cultured in RPMI medium (RPMI 1640 with 10% FCS, L-glutamine and Pen/Strep) at a concentration of 5×10^6^ cells/ml in the presence of either plate bound anti-CD3 (10 µg/ml, Pharmingen) or medium alone and incubated at 37°C. After 3 days, supernatants were collected and stored at −80°C until further analysis.

Six days after RSV infection, RNA was isolated from lungs using TRIZOL reagent (Invitrogen), according to the instructions of the manufacturer. Each sample was reverse transcribed into cDNA and analyzed by quantitative real-time PCR using Taqman (Applied Biosystems). Raw data were normalized to GAPDH control standards in each sample.

Commercially available Biorad X-plex kits (using luminex technology) were used measure cytokine and chemokine levels in culture supernatants. Mouse IFN-α was measured using a commercial ELISA kit from PBL Biomedical Laboratories which has a detection limit of 13 pg/ml.

### Flow cytometry of lungs and lymph nodes

Lungs and lung draining mediastinal lymph nodes were isolated and dispersed using 0.2% collagenase (Type IV, Sigma-Aldrich) in RPMI with 10% FCS at 37°C for 45 minutes. After lysis of red blood cells and blocking non-specific binding by FcR, cells were counted and stained with antibodies or isotype controls. The following antibodies were used: anti-CD3, anti-CD4, anti-CD8, anti-CD11c, anti-B220, anti-CD11b, anti-CD69 (Pharmingen) and RSV M2 82-90 peptide pentamer (Proimmune). Cells were fixed with paraformaldehyde (1%, overnight) and kept in the dark at 4°C until analysis on a Cytomics FC500 flow cytometer (Beckman-Coulter). For intracellular cytokine staining, lung cell suspensions were cultured at a concentration of 10^6^ cells/ml medium in 24 well plates for 4 h in the presence of PMA (50 ng/ml), ionomycin (500 ng/ml) (Sigma) and Golgi stop (1 µl/ml, Becton Dickinson). Following fixation and permeabilization, cells were stained with anti-IFN-γ (Pharmingen) or isotype control.

### Statistical analysis

Differences between groups were analyzed by using one-way ANOVA with a bonferrroni post-hoc test. With a p value of <0.05, differences were considered significant.

## Results

### RSV infection induces differential responses in pDC and cDC

**Figure 1:**
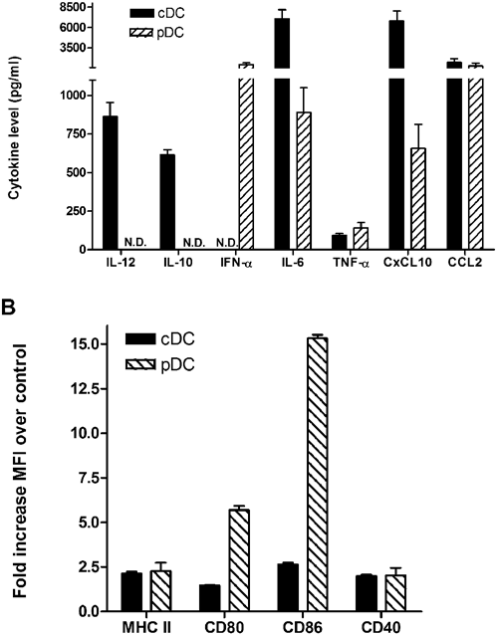
Differential activation profiles of pDC and cDC by RSV. Bone marrow from BALB/c mice was cultured with Flt3L or GM-CSF for preferential expansion of pDC and cDC, respectively, as described. Isolated cell populations (1.10^6^/ml) were subjected to RSV infection (MOI-2.0) for 24 hrs and the supernatants and cells were collected to assess cytokine levels by luminex (A) and surface activation markers by flow cytometry (B). The mean fluorescent intensity (MFI) was assessed using the entire cell population in control vs. RSV-infected cells. The data represent mean±SEM of 4 repeat experiments.

Because cDC and pDC populations have been implicated in anti-viral responses, our initial studies examined the differential expression of immune mediators after RSV infection. Using isolated cDC (CD11b+/CD11c+) and pDC (B220/CD11c+) populations cultured from bone marrow of naive mice various cytokines were measured after RSV infection (MOI = 2.0). The data presented in Figure 1A demonstrate that the two DC subsets differentially produced cytokines in response to a RSV infection. Conventional DC produced IL-10, IL-12, IL-6 and CxCL-10 with no detectable IFN-α, while the pDC produced high levels of IFN-α, lower levels than cDC of IL-6, CxCL10 and no detectable levels of IL-10 and IL-12. Both DC subsets were able to produce similar levels of TNF-α and CCL2. When activation markers were examined by flow cytometry, there were also significant differences in the increase in expression of costimulatory markers, CD80 and CD86, as the pDC more significantly upregulated those two molecules compared to the cDC subset (Figure 1B). Both subsets similarly upregulated class II and CD40 after RSV infection.

### Selective expansion of the number of cDC and pDC in the lung by Flt3L treatment and 120G8

**Figure 2:**
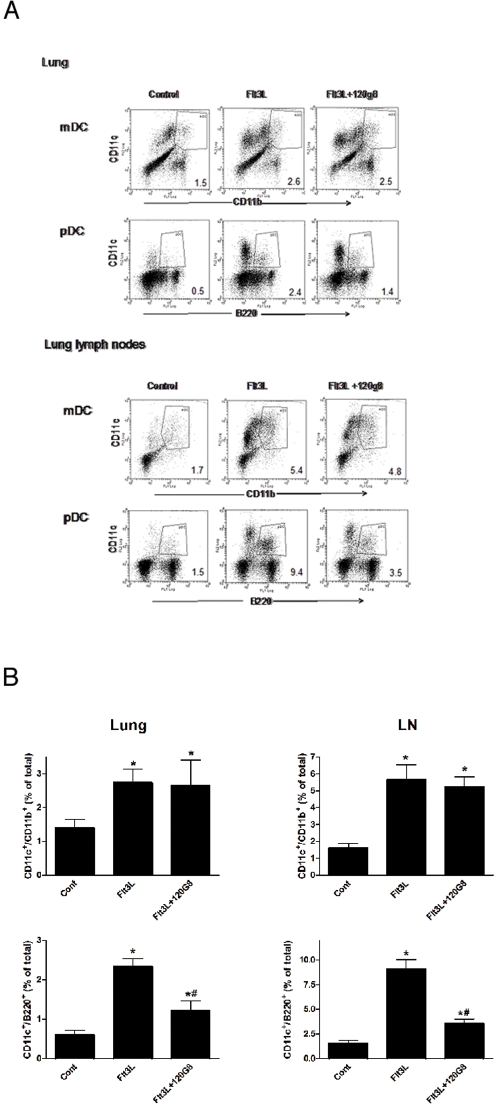
Selective expansion of cDC and pDC in the lung and the lung draining lymph nodes by Flt3L and 120G8 treatment. After Flt3L and 120G8 treatment, lung and lung lymph node cell suspensions from control, Flt3L treated (Flt3L) and pDC depleted Flt3L treated (Flt3L+120G8) mice were analyzed by flow cytometry for the presence of pDC and cDC (mDC). Numbers indicate the percentage of total cells in the gate shown. Shown are the representative flow plots of 3 experiments (A). The mean±SE from all of the mice in 3 experiments demonstrates that only pDC have been depleted in the lung and lymph node (LN) with the 120G8 (B).

We subsequently studied the functional role for cDC and pDC *in vivo*. Our previously published data demonstrated that by depletion of the pDC subset we were able to significantly alter the immune response to RSV *in vivo* [14]. Now, studies were designed to extend those earlier observations in order to better define the role of the individual subsets of DC during the pulmonary immune response. To expand the number of pDC and cDC *in vivo*, mice were treated with Flt3L for 10 days as previously described [11]. Lungs and lung draining lymph nodes were analyzed after this treatment for the presence of CD11c^high^/CD11b^high^ cDC or CD11c^+^/B220^+^, pDC (Figure 2). These data show that Flt3L treatment dramatically increased the number of both pDC and cDC in the lung and lung draining lymph nodes of naïve, uninfected mice. To study the impact of increased numbers of cDC on subsequent development of RSV responses, mice with Flt3L treatment were given the pDC-depleting antibody 120G8, which inhibited the increase of pDC but not cDC. Although this antibody did not completely remove all pDCs, the treatment significantly reduced pDC numbers while allowing the increased numbers of cDC to be maintained. No effect of Flt3L treatment or pDC depletion was observed on the number of CD4+, CD8+ T cells, DX5+ NK cells or CD19+ B cells in the lungs of naïve mice (data not shown). The combined data from all of the treated mice is summarized in Figure 2B and confirms that 120G8 significantly reduces only pDC and not cDC subsets. Conclusively, this protocol provides an avenue for investigating whether increased pDC or cDC numbers could modulate the response to RSV.

### DC expansion attenuates RSV-induced immunopathology, dependent upon pDC

**Figure 3:**
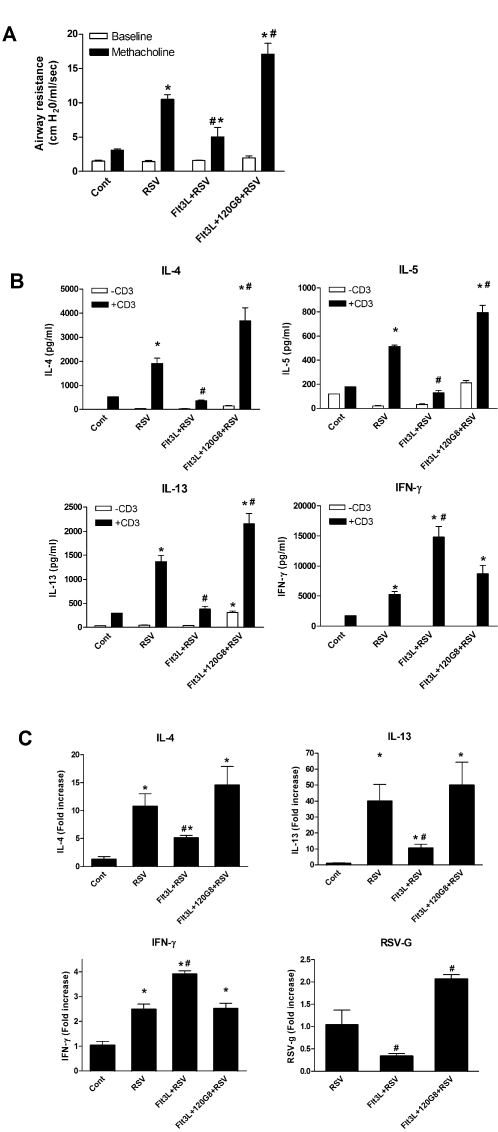
Selective expansion of cDC and pDC affects RSV-induced airway hyperresponsiveness and immunological responses in the lung. (A) Eight days after RSV infection, airway responses were measured in control (cont), RSV-infected (RSV), Flt3L treated and RSV-infected (Flt3L+RSV) and Flt3L treated, pDC depleted, RSV infected (Flt3L+120G8+RSV) mice after one dose of methacholine *(125 mg/kg)* and compared to basal measurements. Data are represented as mean airway resistance in cm H_2_O/ml/sec±SEM. (B) Lung lymph node T cell cytokine production in response to anti-CD3 stimulation. Eight days after infection, lung lymph nodes were isolated from control (Cont), RSV-infected (RSV), Flt3L treated and RSV-infected (Flt3L+RSV) and Flt3L treated, pDC depleted, RSV infected (Flt3L+120G8+RSV) mice and stimulated with anti-CD3 at a concentration of 5×10^6^ cells/ml. Data are represented as mean ng/ml±SEM. (C) Cytokine messenger RNA levels in the lungs of mice. Six days after infection, mRNA was isolated from lungs of control (Cont), RSV-infected (RSV), Flt3L treated and RSV-infected (Flt3L+RSV) and Flt3L treated, pDC depleted, RSV infected (Flt3L+120G8+RSV) mice and analyzed using quantitative real-time PCR by Taqman. Each sample was normalized using a GAPDH control and the data show average fold increase to control±SEM. * *P*<0.01 compared to non-infected control mice, # *P*<0.05 compared to RSV infected mice, n = 5 mice per group. This experiment was repeated twice with similar numbers of mice and displayed comparable results.

We next investigated whether the selective expansion of cDC and pDC, or cDC alone, affected manifestations of RSV-induced immunopathology in the lung. RSV-infected mice developed airway hyperreactivity, as measured by a strong increase in airway resistance in response to methacholine compared with control mice on day 9 (Figure 3A). Expansion of both cDC and pDC by Flt3L diminished this airway hyperreactivity. In contrast, expansion of cDC alone significantly enhanced the airway hyperreactivity induced by RSV. Next, we studied effects of DC expansion on the cytokine environment in the lung draining lymph nodes and the lung itself. RSV infection enhanced production of both the Th1 cytokine IFN-γ and the Th2 type cytokines, IL-4, IL-5, and IL-13 by lymph node T cells after restimulation compared to naïve mice (Figure 3B). Expansion of both cDC and pDC significantly inhibited IL-4, IL-5 and IL-13 production, but enhanced IFN-γ production. In contrast, when pDC were depleted a significant enhanced IL-4, IL-5 and IL-13 production was observed, while IFN-γ production remained the same as in the RSV control animals. In order to analyze cytokine expression in the lung, real-time PCR analysis of the lung showed that RSV infection elicited transcription of IL-4, IL-13 and IFN-γ in the lung (Figure 2C). Again, expansion of both DC subtypes lowered transcription of IL-4 and IL-13 and enhanced IFN-γ, while expansion of cDC alone significantly enhanced the transcription of IL-4 and IL-13 and lowered IFN-γ expression to normal levels. In addition, analysis of expression of RSV G protein in the lung showed that expansion of cDC and pDC inhibited expression of RSV in the lung, while expansion of only cDC significantly enhanced expression of RSV (Figure 3C).

**Figure 4:**
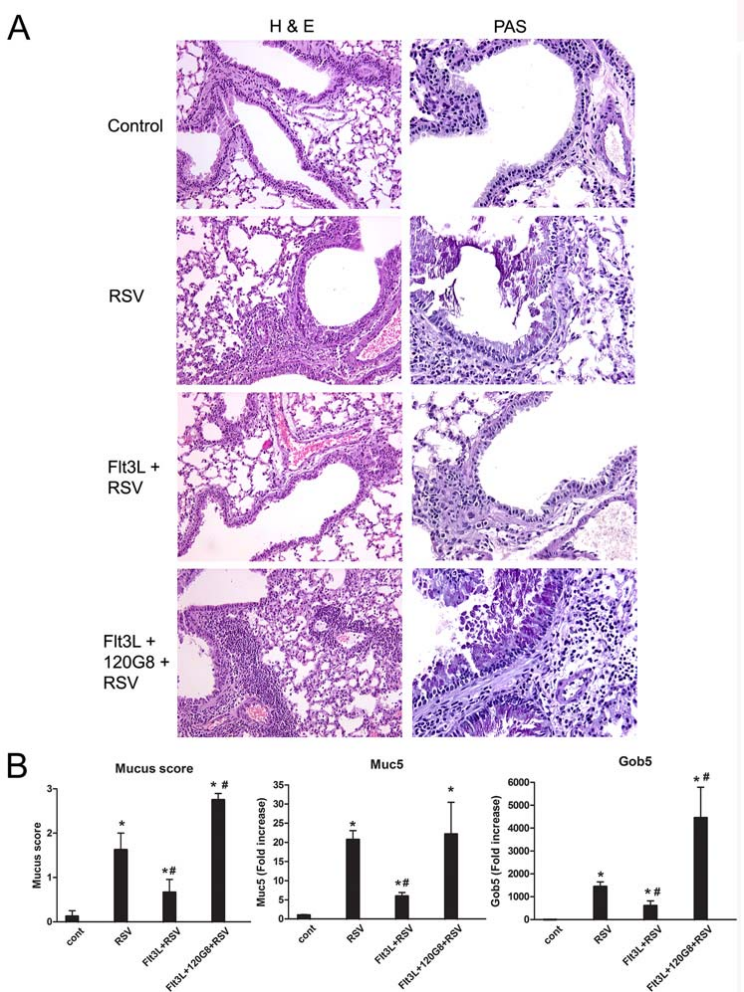
RSV induced-inflammation and mucus production in the lung is affected by selective expansion of cDC and pDC. (A) Eight days after infection, lung sections were stained with H&E and PAS. Shown are representative sections of control (Cont), RSV-infected (RSV), Flt3L treated and RSV-infected (Flt3L+RSV) and Flt3L treated, pDC depleted, RSV infected (Flt3L+120G8+RSV) mice in a 200× (H&E) or 400× (PAS) magnification. (B) To quantify the mucus production in the lung, PAS sections were randomized, examined in a blinded fashion and scored on a scale from 1 to 4, with 1: representing no mucus cell content, 2: one airway with PAS staining, 3: Multiple airways with strong PAS staining, 4: Multiple airways with strong PAS staining and mucus filled airways. In addition, RNA was isolated from the lungs of control (Cont), RSV-infected (RSV), Flt3L treated and RSV-infected (Flt3L+RSV) and Flt3L treated, pDC depleted, RSV infected (Flt3L+120G8+RSV) mice and transcribed into cDNA. Samples were analyzed for Muc5ac and GOB-5 mRNA levels using quantitative real-time PCR by Taqman. Each sample was normalized using a GAPDH control and the figure shows average fold increase to RNA obtained before infection±SEM. * P<0.05 compared to control mice, # P<0.05 compared to RSV infected mice, n = 5–6 mice.

Histological examination of control mice showed that RSV infection leads to a peribronchial and perivascular inflammatory infiltrate consisting of mononuclear cells and leads to goblet cell hyperplasia and mucus production in the lung (Figure 4A). After expansion of cDC and pDC animals appeared less inflamed and displayed much less intense goblet cell hyper/metaplasia (Figure 4B), as assessed using a blinded subjective scoring system. Assessing 2 mucus-associated genes, muc5ac and gob5 supported the subjective scoring analysis showing a decrease in the expression of both genes after expansion of cDC and pDC but an increase after pDC depletion (Figure 4B).

### Modulation of the number of cDC and pDC in the lung after RSV infection by Flt3L and the 120G8 antibody

**Figure 5:**
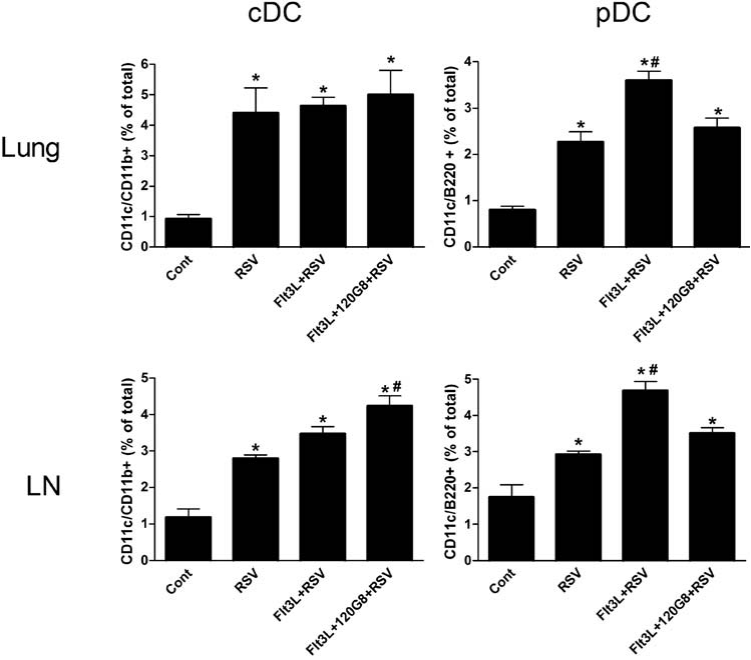
Selective expansion of cDC and pDC before RSV infection affects the number of cDC and pDC after RSV infection. Six days after RSV infection, single cell suspensions of lungs and lung draining lymph nodes (LN) from control (Cont), RSV-infected (RSV), Flt3L treated and RSV-infected (Flt3L+RSV) and Flt3L treated, pDC depleted, RSV infected (Flt3L+120G8+RSV) mice were analyzed by flow cytometry for the presence of cDC (CD11c^high^/CD11b^high^) or pDC (CD11c^+^/B220^+^). Results are displayed as precentage of total lung cells. * P<0.05 compared to non-infected control mice, # P<0.05 compared to RSV infected mice, n = 3–6 mice per group.

We next investigated whether the selective expansion of DC was maintained in the lung and lung lymph nodes throughout infection. Six days after infection with RSV, numbers of both cDC and pDC were increased in both lungs and lymph nodes (Figure 5). The number of cDC remained elevated irrespective of previous DC expansion. In the lymph nodes however, cDC numbers were significantly higher in Flt3L and 120G8 treated mice. The number of pDC was significantly higher in Flt3L treated mice, which was significantly inhibited by earlier pDC depletion. These data suggest that the initial expansion of pDC is maintained in the lung and lung lymph nodes throughout infection with RSV whereas the initial selective expansion of cDC is only maintained in the lymph nodes. Therefore, it can be concluded that a shift in the balance between cDC and pDC frequency is maintained during a pulmonary infection and may be crucial for the outcome of this infection.

### Expansion of both cDC and pDC increases CD8+ T cell responses to RSV, dependent on pDC

**Figure 6:**
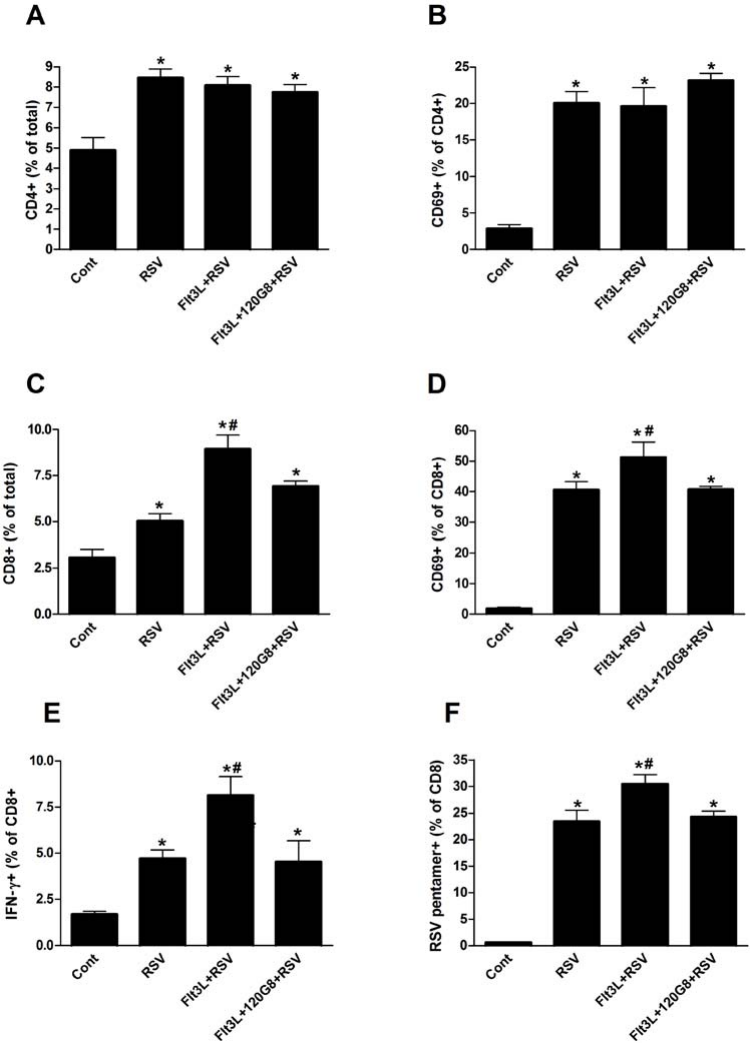
Selective expansion of cDC and pDC affects CD8+ T cell responses but not CD4+ T cell responses in the lung after RSV infection. Six days after infection, single cell suspensions of lungs from control (Cont), RSV-infected (RSV), Flt3L treated and RSV-infected (Flt3L+RSV) and Flt3L treated, pDC depleted, RSV infected (Flt3L+120G8+RSV) mice were analyzed by flow cytometry for the presence or expression of (A) CD3+CD4+ T cells, (B) CD69 on CD4+ T cells, (C) CD3+CD8+ T cells, (D) CD69 on CD8+ T cells, (E) Intracellular IFN-γ expression in CD8+ T cells or (F) RSV M2 MHCI pentamer expression on CD8+ T cells. * P<0.05 compared to non-infected control mice, # P<0.05 compared to RSV infected mice, n = 4–6 mice per group.

The alteration of the inflammatory response and immunopathology has previously been linked to the nature of the T cell activation [21]. To further define the responses after DC expansion by Flt3L, T cell accumulation within the lungs was characterized. Surprisingly, the number of total or activated (CD69+) CD4+ T cells that were elicited to the lungs of RSV infected mice did not change when cDC and pDC or cDC alone were expanded before infection (Figure 6A, 6B). In contrast, the number of CD8+ T cells was significantly increased in Flt3L treated mice that was reduced again when the pDC were depleted (Figure 6C, 6D). Furthermore, additional markers of CD8+ T cell activation showed that expansion of cDC and pDC increased the expression of IFN-γ, assessed by intracellular staining, and increased the percentage of RSV M2 protein specific pentamer positive CD8+ T cells (Figure 6E, 6F). The increase in both of these markers correlates nicely to the decreased pathology. Likewise, when pDCs were depleted in these mice, there was a significant reduction in those CD8 T cells that express IFN-γ and stained positive with the RSV M2 protein specific pentamer. Together, these studies indicate that while expansion of DC had little effect on the number of CD4+ T cells, it significantly enhanced the CD8+ T cell numbers and CD8 effector response. This increased CD8 response was dependent upon the increase in pDC.

## Discussion

Previous studies have demonstrated the utility of using flt3 ligand and described its function for enhancing the innate immune responses through the expansion of DC subsets [10]–[12], [22]–[25]. *In vitro* it is known that flt3 ligand can induce the maturation of cDC, but unlike GM-CSF also induces the differentiation of pDCs. The present studies verify these observations by demonstrating expansion of cDC and pDC in the lungs of naive animals treated with flt3 ligand. The objectives of these studies were to not only expand the DC populations for enhanced anti-viral responses, but also to examine whether the altered immune response was dependent upon the pDC subset. Although the 120G8 did not completely remove all pDCs, the treatment significantly reduced pDC numbers while allowing the increased numbers of cDC to be maintained and respond to the virus infection. These data point out several important aspects. Firstly, the results verify the importance of pDC during viral infections for promoting the appropriate and non-pathogenic responses. Secondly, it indicates that providing more cDCs within the lung is not by itself sufficient for enhancing the anti-viral responses. Thirdly, it suggests that pDC have a role in enhancing CD8 T cell maturation and/or differentiation during the viral responses. This latter issue may be both direct and indirect through APC function and type I IFN production. While these studies allow a better understanding of the relative role of the pDC subset functions during RSV infection, they do not fully define how the individual subsets contribute. In fact, numerous studies have offered data suggesting that one important role of pDCs during anti-viral or anti-tumor immunity may be for shaping the utility of the cDC for proper APC function [7], [8], [26], [27]. By examining the cytokine profiles of the two DC subsets in response to RSV a picture might be drawn on how these two subsets differentially contribute to the immune phenotype. While the cDC are capable of producing significant levels of IL-12 that would enhance IFNγ production from T and NK cells, the pDC preferentially produces type I IFN that may augment NK cell and CD8 T cell differentiation into a cytotoxic phenotype [28]. Interestingly, our previous studies demonstrated that depletion of pDC in normal mice led to an increase in IFNγ production, whereas in this study with Flt3L treatment the reduction in pDC led to a decrease in IFNγ. While we have not explored this further, one explanation may be the relationship to the fluctuation in the CD8 T cell population displayed in Figure 6 related to the Flt3L treatment. Related to this latter issue, the observation that the RSV infection significantly upregulated CD80 and CD86 expression on the pDC may also contribute to this observation. A recent study demonstrated using animals devoid of cDC subsets that pDC could participate in APC function in lymph nodes for CD4 T cell activation [29], a notion suggested by other previous studies *in vitro* [30], [31]. Together, this may suggest a direct or complimentary role for pDC, along with the cDC, to function as APCs locally and/or in the lymph node during RSV infection and enhance the anti-viral responses, including IFNγ production.

Classically, pDC are considered the primary source of IFN-α. Although type I IFN can impact numerous cells involved in the immune response [32]–[34], our previous reconstitution studies with type I IFN after pDC depletion did not demonstrate an effect on the nature of the immune response [14]. Other recent studies have also indicated that cellular contact was necessary for pDC to influence cDC ability to alter T cell responsiveness and soluble factors were not sufficient [7]. Thus, we are presently considering a reasonable model that includes both cDC and pDC interaction with T cells in order to initiate the proper immune response. It is unclear from our present data whether the altered CD8 T cell responses are a direct result of reduced pDC participation or from the altered CD4 T cell cytokine response that would normally influence the differentiation of the cytotoxic T cells. Although it is unclear what the necessary profile of molecules from pDC signal cDC to instruct anti-viral T cell responses, they may include direct interaction with the T cells via CD80 and CD86 upregulated on pDC after RSV infection. Altogether, these data clearly demonstrate the importance of pDC for directing the type of response to RSV. Expansion of both pDC and cDC resulted in a decrease in type 2, but an increase in type 1 T cell response to RSV and to a general lower immunopathology after RSV infection. In contrast, removal of pDC and expansion of only cDC significantly enhanced Th2 type responses to RSV and led to more inflammation and a higher airway response to RSV. As suggested also by others [6], this demonstrates the highly immunogenic role of cDC and the more regulatory role of pDC in the lung.

The coordination of the pulmonary immune response to a viral infection appears to be primarily controlled in part by the presence of the proper APC subsets. Previous studies have defined that specific DC subsets can skew the immune response. Using defined antigen models, early studies defined the nature of the local cDC subset for preferential skewing toward Th2 responses [35]–[38]. However, these latter studies were performed in the absence of pDC populations that were subsequently shown to regulate the development of Th2 responses in lung [6]. The regulation and coordination of the response by these two subsets may indeed depend upon how they recognize a pathogen or antigen. The cDC subset preferentially expresses a specific profile of TLRs that are relevant to RSV infection, including TLR3 (dsRNA) and TLR4 (F protein of RSV) [39], [40]. In contrast, pDC preferentially expresses TLR7 (ssRNA) that would promote type I IFN production [34]. In our own studies with RSV we have found that in the absence of TLR3 there is a skewed response toward increased IL-13 and mucus overproduction, while deletion of MyD88 led to a fully Th2 skewed system that appeared to depend upon IL-12 and other Th1-mediated responses associated with cDC populations [41], [42]. Thus, multiple pathways and cell types are necessary for the most appropriate non-pathogenic response in the lung. Altogether, these studies represent additional evidence that the pDC population can provide additional protection from adverse pathologic outcomes and further support the use of Flt3 ligand as an effective mediator to alter the pulmonary immune response.

Future vaccine strategies for RSV should focus on the role of pDC during the immune response as well as a balance between cDC and pDC in the lung and draining lymph nodes that seems to be crucial for the outcome of a pulmonary infection. One limitation of using Flt3L for expansion of pDC subsets during specific diseases will be when and in what situations will it be most relevant to use such a treatment modality. Finally, these studies represent additional evidence that the pDC subset can initiate an environment that is protected from adverse pathologic outcomes during a pulmonary immune response.
